# The Passions in Their Relation to Health and Disease

**Published:** 1873-08

**Authors:** 


*The Passions in their relation to Health and Disease.* Translated from the French of Dr. X. Bourgeois. By Howard F. Damon, A. M., M. D. Boston: Jas. Campbell, 1873.

The author of this work, if we take the title as a guide, proposes to speak of the relation of the Passions to Health and Disease. He divides his production into two parts: Love and Libertinism, and then treats his reader with a review of some of the most disgusting phases of Parisian life. We see nothing in the work to recommend it to the reader and much to condemn it. In fact did it not receive the sanction of an American Physician in good standing, whose name we are sorry to see connected with it, we should look upon it as the advertisement of a quack worthy only of being classed with the productions of those who make their capital upon the judicious use of the phrases "Early indiscretion," etc., etc.

This is designed for popular reading and with all its faults, it states many facts connected with the reproductive functions of poor humanity which the public will be interested in. Indeed, it is a book which will be read, it has been written to be read and nothing can suppress the reading of this and all similar books. That any one will be much the wiser or any better for its instructions is more doubtful.

